# Direct Determination of the Mutation Rate in the Bumblebee Reveals Evidence for Weak Recombination-Associated Mutation and an Approximate Rate Constancy in Insects

**DOI:** 10.1093/molbev/msw226

**Published:** 2016-10-20

**Authors:** Haoxuan Liu, Yanxiao Jia, Xiaoguang Sun, Dacheng Tian, Laurence D. Hurst, Sihai Yang

**Affiliations:** 1 State Key Laboratory of Pharmaceutical Biotechnology, School of Life Sciences, Nanjing University, Nanjing, China; 2 Department of Biology and Biochemistry, The Milner Centre for Evolution, University of Bath, Bath, United Kingdom

**Keywords:** mutation rate, recombination rate, biased gene conversion, bee

## Abstract

Accurate knowledge of the mutation rate provides a base line for inferring expected rates of evolution, for testing evolutionary hypotheses and for estimation of key parameters. Advances in sequencing technology now permit direct estimates of the mutation rate from sequencing of close relatives. Within insects there have been three prior such estimates, two in nonsocial insects (*Drosophila*: 2.8 × 10^−^^9^ per bp per haploid genome per generation; *Heliconius*: 2.9 × 10^−^^9^) and one in a social species, the honeybee (3.4 × 10^−^^9^). Might the honeybee’s rate be ∼20% higher because it has an exceptionally high recombination rate and recombination may be directly or indirectly mutagenic? To address this possibility, we provide a direct estimate of the mutation rate in the bumblebee (*Bombus terrestris*), this being a close relative of the honeybee but with a much lower recombination rate. We confirm that the crossover rate of the bumblebee is indeed much lower than honeybees (8.7 cM/Mb vs. 37 cM/Mb). Importantly, we find no significant difference in the mutation rates: we estimate for bumblebees a rate of 3.6 × 10^−^^9^ per haploid genome per generation (95% confidence intervals 2.38 × 10^−^^9^ and 5.37 × 10^−^^9^) which is just 5% higher than the estimate that of honeybees. Both genomes have approximately one new mutation per haploid genome per generation. While we find evidence for a direct coupling between recombination and mutation (also seen in honeybees), the effect is so weak as to leave almost no footprint on any between-species differences. The similarity in mutation rates suggests an approximate constancy of the mutation rate in insects.

## Introduction

Accurate estimation of the mutation rate is necessary for establishing a base rate for molecular evolution in the absence of biased gene conversion or natural selection. In principle, if coupled with heterozygosity data, it also affords the possibility of estimation of effective population size (Keightley et al. 2014). In addition, understanding the rate of new mutations that are deleterious is a key parameter for evolutionary hypotheses, such as the mutational deterministic model for the evolution of sex (Kondrashov 1988). Mutation rate estimation is however problematic. One can calculate rates from between-species comparisons (EyreWalker and Keightley 1999; Keightley and Eyre-Walker 2000; Nachman and Crowell 2000) but these require assumptions of effective neutrality of analyzed sites (Chen and Zhang 2013) and accurate estimation of the generation time. Accumulation of mutations through mutation accumulation lines has been an increasing popular method (e.g. Ossowski et al. 2010; Sharp and Agrawal 2016), but also comes with difficulties of selection removing some mutations and with assumptions needed to infer a per generation rate. With recent advances in sequencing technology it is now possible to provide relatively assumption-free direct estimation of the mutation rate via parent-offspring sequencing with stringent filters to avoid false positive calls (Roach et al. 2010; Conrad et al. 2011; Kong et al. 2012; Michaelson et al. 2012; Keightley et al. 2014, 2015; Yang et al. 2015).

This direct estimation has been attempted to date for three species of insect (Keightley et al. 2014, 2015; Yang et al. 2015). In the two nonsocial species (*Heliconius melpomene*, Keightley et al. 2015 and *Drosophila melanogaster*, Keightley et al. 2014), the estimates are remarkably similar at 2.9 × 10^−^^9^ (with an upper 95% limit of 5.5 × 10^−^^9^) and 2.8 × 10^−^^9^ per base per haploid genome per generation (with an upper 95% limit of 6.1 × 10^−^^9^), respectively. We recently reported, via whole genome sequence in honeybees over one generation, both an estimate of this species’ recombination rate (Liu et al. 2015) (37 cM/Mb) and mutation rate (Yang et al. 2015) (6.8 × 10^−^^9^ per diploid genome in a diploid queen per generation, hence 3.4 ×10^−^^9^ per haploid genome from queen to haploid drone). We note that the honeybee mutation rate may be a little higher (∼20%) than that seen in the two nonsocial species, although the 95% limits contain the lower rates (95% confidence interval for honeybees= 2.2 × 10^−^^9^ ∼ 4.9 × 10^−^^9^). Nonetheless, given the exceptionally high recombination rate in honeybees (typical of social insects; Wilfert et al. 2007), this led us to hypothesize that perhaps honeybees might have a higher mutation rate because they have an exceptionally high recombination rate.

Such a conjecture is not without precedent, not least because there is a suggestion that recombination is directly mutagenic (Magni and von Borstel 1962; Magni 1964), possibly owing to error prone double strand break repair. The hypothesis has attracted a body of both supportive (Perry and Ashworth 1999; Lercher and Hurst 2002; Filatov and Gerrard 2003; Huang et al. 2005) and unsupportive (Aquadro et al. 2001; Nachman 2001; Betancourt and Presgraves 2002; Duret and Arndt 2008; Abecasis et al. 2010) results. Much of the supportive evidence (Perry and Ashworth 1999; Lercher and Hurst 2002; Filatov and Gerrard 2003; Huang et al. 2005) derives from a correlation between substitution rates and recombination rates. This now appears to be more parsimoniously explained as a consequence of biased gene conversion (Duret and Arndt 2008), which indeed explains why the effect on substitution rates is weak compared with the effects on GC content (Huang et al. 2005). Nonetheless, recent more direct experimental high-resolution data suggests a mutagenic effect of recombination in diverse taxa (Pratto et al. 2014; Arbeithuber et al. 2015; Rattray et al. 2015; Yang et al. 2015). If true we expect to see an excess of new mutations in the vicinity of recombination break points.

**Fig. 1:**
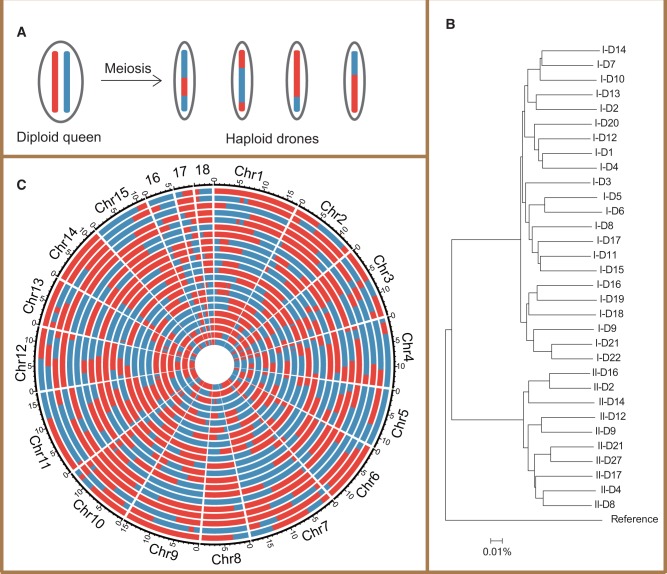
Relationship between queen and drones, and recombination map of drones. (*A*) Schematic description of the queen–drone relationship within a colony. (*B*) Polygenetic relationship of the drones sequenced in this study. The phylogenetic tree was constructed using the polymorphic sites across the whole genome between drones based on the bootstrap Neighbor-Joining method with number of differences model by MEGA (Tamura et al. 2013) v6.0. The reference genome was used as an outgroup. (*C*) Map of recombination of 22 drones in colony I. The two haplotypes of the queen are represented by red and blue, each circle represents a drone.

To address the above hypothesis and, more generally the possibility that honeybees may have a high mutation rate because they have a high recombination rate, we now estimate the mutation rate in the honeybees’ close relative, the relatively primitively social bumblebee (*Bombus terrestris*), by deep sequencing the whole genome of 32 drones, 22 of them from a same queen defined as colony I, 10 of them from another queen as colony II (fig. 1*B* for relationships). Both bumblebees and honeybees are members of the monophyletic Corbiculates (Kawakita et al. 2008; Cardinal et al. 2010) with an age to common ancestry estimated at *circa* 80MY (Cardinal and Danforth 2011). Importantly, bumblebees have a very much lower recombination rate (Stolle et al. 2011). The prior estimate (Stolle et al. 2011) for the recombination rate of 4.76–8.19 cM/Mb (compared with 37 cM/Mb in honeybees; Liu et al. 2015) was conditioned on the then unknown size of the bumblebee genome. The higher estimate assumed a size of 250 Mb, quite close to the actual 274 Mb (Sadd et al. 2015). Assuming a size of 274 Mb, the prior map distance of 2047.09 cM (Stolle et al. 2011) suggests a rate of 7.47 cM/Mb. Thus *prima facie*, these two closely related species have almost 5-fold different recombination rates. In part this difference likely reflects differences in marker density, the high honeybee estimates being derived from an analysis with exceptionally high density (314-bp interval between adjacent markers)(Liu et al. 2015). Indeed, lower marker density estimates for the honeybee (∼100-kb interval) derives an estimate of circa 19 cM/Mb (Beye et al. 2006). Thus for fairer comparison of the difference between bumblebee and honeybee we need estimates based on comparable marker densities.

Bees come with unusual advantages for mutation and recombination rate analysis and not just because both bee genomes are now well described (Weinstock et al. 2006; Sadd et al. 2015). Bee queens lay both fertilized and nonfertilized eggs. Fertilized eggs develop into diploid workers, whereas nonfertilized eggs develop into haploid drones (fig. 1*A*). The haploid nature of the drones obviates experimental difficulties associated with heterozygosity, making inference of mutation and recombination by next-generation sequencing more accurate and straightforward. As we are analyzing the mutation rate via close relative sequencing, we can simultaneously examine the hypothesis that recombination may be mutagenic, as we can determine the location of new mutations and recombination events in the self same individual (as opposed to looking for general correlations between mean recombination rates and mean mutation rates summed over many individuals).

Here then we ask the following: (1) what is the mutation rate of the bumblebee, (2) is there any evidence that it is lower than that of the honeybee, (3) are there more mutations in close proximity to recombination breakpoints than expected, (4) if they do, might this explain the possibly raised mutation rates in honeybees? In addition, we provide a fine scale resolution recombination map of the bumblebee genome and ask whether we can confirm a difference in the recombination rate, when controlling for marker density. The same analysis permits us to detect signals of biased gene conversion. We report that the two bee species have almost exactly the same mutation rate while confirming a much reduced recombination rate in bumblebees. While we find a very small excess of new mutations in the immediate vicinity of recombination events, the effect is so weak as to be almost out of the range of our analysis and not an important contributor to between-species differences. We provide support for the view that recombination-associated biased gene conversion operates in this species associated with noncrossover events. We cannot exclude the hypothesis that all insects, regardless of sociality or recombination rate, have approximately the same mutation rate.

## Methods

### Sample Source, DNA Extraction and Genome Sequencing

The bumblebee queens were obtained from a pollination company (http://www.sdfd.net/; last accessed October 19, 2016). Each bumblebee queen was reared individually in a confined bumblebee nest under standard conditions (Röseler 1985) (temperature: 25∼30°C; humidity: 50%∼80%). They were not artificially inseminated. Since drones are the parthenogenetic product of their mother queen, the number of matings per queen is irrelevant. The bumblebees were fed with honey and pollen weekly. After 3∼4 months, drones start to emerge in each colony. The drones from two colonies were collected for this study. Usually progeny drones and queens emerge at almost the same time. It is hard to identify the colony queen from its progeny queens. Therefore, the colony queens were not used in this study. Moreover, as marking queens prior to reproduction might prove stressful and stress may well affect the mutation rate (Sharp and Agrawal 2016), it is possibly optimal not to mark and sequence queens if this is unnecessary. Mutations can nonetheless be called by reference to the orthologous site in the full sibs. Thus this study is not formally parent-offspring sequencing but is expected to have the same degree of rigor. Phylogenetic evidence (fig. 1*B*) supports the close relatedness of drones within each colony, indicating that none are worker derived.

The DNA of each drone was individually extracted using phenol/chloroform/isoamyl alcohol method. The abdomens of drones were removed before DNA extraction to avoid microbial contamination. Construction of the DNA libraries and Illumina sequencing were performed at BGI-Shenzhen as the following procedure: Paired-end sequencing libraries with insert size of 350 bp were constructed for each drone, then 2 × 150 bp paired-end reads were generated on Illumina HiSeq 4000 platform. The average read depth for each sample is over 26× (supplementary table S1, Supplementary Material online), the sequencing reads are available on NCBI under accession number SRP076825.

### SNP Calling, Marker Identification, Mutation Identification

Bumblebee (*B. terrestris*) reference genome (v 1.0) was downloaded from NCBI (ftp://ftp.ncbi.nlm.nih.gov/genomes/Bombus_terrestris/; last accessed October 19, 2016). The sequencing reads were mapped onto the reference genome by BWA (Burrows-Wheeler alignment tool, Li and Durbin 2009) then duplicates marking and realignment around indels were performed by Genome Analysis Toolkit (GATK) (McKenna et al. 2010) with variants called by GATK HaplotypeCaller.

SNPs called in drones were used as markers and screened by the following procedure: (1) as drones are haploid each SNP must called as “homozygous” in each drone. The “heterozygous” SNPs were removed due to potential mapping errors or copy number variations (41); (2) For each marker site, only two genotypes can be identified in the drone cohort from the same colony; (3) The candidate markers must be called with high sequence quality (≥30) in ≥90% samples. Finally, 553,969 (0.27%) and 508,013 (0.25%) accurate SNP markers were identified in colony I and II, respectively. The genotypes of all samples at all marker sites are available at http://gattaca.nju.edu.cn/pub_data.html (last accessed October 19, 2016).

The specific SNPs and small indels (<20 bp) were identified in each drone as mutation candidates (supplementary fig. S1, Supplementary Material online), then these SNPs were screened by the following criteria: (1) Read depth ≥5 and quality score ≥30; (2) The candidate locus is covered with both forward reads and reverse reads; (3) The candidate cannot be called in all of the other drones; and (4) Finally, we performed manual examination for mis-alignment or mis-call. We screened out 8 candidate point mutations and 16 candidate indels via this manual screening.

To calculate the mutation rate, the total number of mutations was divided by combined number of callable sites from all samples. Callable sites are defined as those with read depth ≥5 and mapping quality ≥20 and no ambiguous bases in reference genome. To estimate the false negative rate and to verify the number of callable sites, we applied a method that introduces false mutations (in silico) into the fastq output files (Keightley et al. 2014). We generated 10,000 single point substitution mutations randomly across the whole genome. Since drones are haploid, the mutations we generated are homozygous. Out of these 10,000 synthetic mutations, 91.71% (9,171) are callable, which is similar to our estimation that 92.61% of the genome is callable. Of these 9,171 callable synthetic mutations, 99.8% (9,157) were identified as positive mutations through our pipeline.

To estimate the false positive rate, we performed Sanger sequencing on all of the 25 mutations identified in this study (23 SNPs and 2 indels). For each putatively mutated locus, the mutated sample and 3∼5 nonmutated samples were selected for PCR and Sanger sequencing. About 24 of the 25 mutated loci sequenced successfully (one failed in the PCR process). Of these 24 that we could sequence, all were detected only in the mutated samples and not in the nonmutated sample. Thus, as before with this method (Yang et al. 2015) the false positive rate is negligible.

### Haploid Phasing and Identification of Recombination Events

In each colony, all of the identified markers were used for haploid phasing. In practice, for each and every two adjacent markers, if the genotypes of the two markers are linked in most drones, these two genotypes are assumed to be linked on the same chromosome in the queen. For example, assuming two adjacent markers being “A/C” and “T/G”, there could be two types of linkage in their mother queen, “A-T, C-G” or “A-G, C-T” (For missing calls in one or two samples, the linkage is inferred from the other samples). Due to the low probability of recombination events per unit physical distance, if more “A-T, C-G” drones are identified than “A-G, C-T” drones, then “A-T, C-G” is assumed to be the correct linkage in the queen. A real example is shown in supplementary figure S2, Supplementary Material online, which shows the genotypes of 22 drones from colony I at 14 marker positions in a ∼7-kb region in chromosome 10. Using this method, the 14 markers are phased into blue and red haplotypes, so resolving the queen’s two haplotypes at the chromosomal level.

When the reconstructed queen haplotypes were obtained, the genotypes of each drone were compared with the queen haplotypes to identify recombination events. As a result, we obtain mosaic drone chromosomes, where genotype blocks change between the two haplotypes of the queen (as shown in fig. 1C). Genotype changes can be the result of either crossover or noncrossover. Here we define, as before (Liu et al. 2015), blocks span ≤10 kb as noncrossovers (NCO), whereas blocks span >10 kb are crossovers (CO). As the great majority of recombination events are in the >100-kb range, relaxing this assumption makes little difference to recombination rate measures (supplementary table S3, Supplementary Material online). In this way, the CO and NCO events in each drone can be identified.

### Analysis of Shared CO Events and Exclusion of Potential Translocations and Mis-Assemblies

With the above methodology, in a complete well-finished genome we do not expect to see many incidences of recombination events shared by more than one drone. An initial analysis identified 734 COs in these 32 drones, 250 of which are shared by more than one drone. One explanation is that some of these 250 are real recombination events, but ones that occur in large gapped regions of the genome build. Two events at different locations in a gapped domain will appear as if they occur at the same site in the two drones. Indeed, a closer inspection found big reference gaps or unmapped regions at the breakpoint intervals of all these events. About 82% of the 250 events are found with big reference gaps (represented by 50,000 “N”s), 18% these events are found with unmapped regions running from 200 bp to 5 kb, which also represent unknown gaps between our samples and the reference genome.

The 250 events can be subdivided into two categories: (1) the shared CO event is the only shared CO on its chromosome; (2) the shared CO event is next to another shared CO event on the same chromosome in the same sample. About 122 of the 250 events belong to the first category, for this category, since the length of these gaps in breakpoint intervals are unknown and the flanking markers were reliable, it is possible that COs occurred in multiple samples in these regions. However, we are cautious with the second category, since translocations and mis-assemblies in the reference genome can lead to false positives for double CO events (two CO events that are close to each other on the same chromosome). The 128 events in the second category are all double COs and shared in drones, the distance of these double COs ranging from ∼100–700 kb. It is highly possible that these 128 COs were introduced by translocations in our strains, or mis-assemblies in the reference genome. Thus these 128 COs were excluded. Finally, 734−128= 606 COs were kept.

### Statistical Analysis

To determine correlates to the recombination rate we divide the bumblebee genome into nonoverlapping blocks of 100 kb. We then calculate the parameters under scrutiny in each such block. We consider gene density (number of genes per bp of sequence), exon percentage (span of exonic coding sequence per base), GC content (percentage of identifiable bases that are G or C) and heterozygosity (proportion of sites heterozygous between the two haplotypes from the same colony, averaged for two colonies in this study). For each analysis we rank order the 100-kb windows. We then merge data from rank ordered neighboring blocks (neighboring in the rankings, not in the genome) into 21–25 blocks. In this method each merged block is of approximately uniform content as regards the parameter in question. We take the average of the data within each of these merge blocks. We then perform regression analysis on the resulting data. Note that this method of merging is helpful in permitting observation of gross trends. However, the meaning of a *P* value in this context is not the same as a *P* value when applied to raw data. We also thus present the raw correlations observed over several block sizes (supplementary table S4, Supplementary Material online). Partial correlations are calculated by “ppcor” package in R, using Spearman’s method.

To estimate confidence intervals of the estimated mutation rate we assume the observed number of mutations is a Poisson variable. We then apply the Poisson.test function in R to estimate confidence intervals, with confidence intervals set to 0.95. We test for assumptions of Poisson distribution via Monte Carlo simulation (described in text). For the Monte Carlo tests the unbiased estimator of the type I error rate (North et al. 2003) is *P* = (*n *+* *1)/(*m *+* *1), where *n* is the count of randomized observations as extreme or more extreme than observed in the real data and *m* the number of randomizations.

## Results

### Marker Identification and Haplotype Phasing

For the 32 drones (22 from colony I, 10 from colony II, fig. 1*B*) the average depth for DNA sequencing is over 26× and covers 98% of the genome (supplementary table S1, Supplementary Material online). The SNPs identified in each colony were used as markers to identify recombination events. After a series of screening processes (Methods for details), we identified 553,969 (0.27%) and 508,013 (0.25%) accurate SNP markers in colony I and II, respectively (The genotype files are available at http://gattaca.nju.edu.cn/pub_data.html; last accessed October 19, 2016). These densities equate to average inter-marker distance of 373 bp in colony I and 407 bp in colony II. These numbers are comparable to the density (314 bp) employed in estimating the recombination rate in honeybees (Liu et al. 2015). These markers in each colony were used to re-construct two sets of chromosome haplotypes of their respective mother queen (Methods for details). By comparing a drone’s genotype with the phased haplotypes of its queen, we can then infer recombination events for each drone (fig. 1*C*).

### Bumblebees Have Much Lower Crossover Rates than Honeybees

While prior evidence strongly indicates that honeybees have much higher rates of recombination than bumblebees, we start by confirming this observation employing analysis with markers at similar levels of densities in the two species. In each colony, the mosaic drone chromosomes can be detected with genotypes switching from one haplotype to the other of the queen (fig. 1*A* and *C*). This can be used to infer COs or NCOs. Overall, we identified 606 COs in the 32 drones (table 1 and supplementary table S2, Supplementary Material online), averaging 19 COs per drone and 1.1 COs per chromosome per drone. We estimate the rate of recombination to be 8.73 cM/Mb [100 cM × 19.44 (COs per drone)/216.85 Mb (combined length of assembled chromosomes)], slightly higher than previous estimates (∼7.5 cM/Mb, 2,047 cM/274 Mb), but much lower than honeybee (37 cM/Mb). Even if we include the 128 dubious “recombination” events, the estimate is only ∼10.6 cM/Mb, still much lower than that of the honeybee.

**Table 1:** Comparison of Recombinational and Mutational Landscape between Bumblebee and Honey Bee.

	Bumblebee	Honeybee
Genome size (Mb)	274	236
No. of chromosomes	18	16
Genome GC-content	37.5%	34.0%
Rate of CO (cM/Mb)	***8.7***	37.0
No. of COs per Chromosome	***1.3***	5.1
No. of NCOs per sample	***0.7***	5.1
No. of markers converted by NCOs per sample	***1.6***	31
Mutation rate	***3.6 × 10^−9^***	3.4 × 10^−9^

Note.—Data for honeybees from Liu et al. (2015), Weinstock et al. (2006), and Yang et al. (2015). Data for bumblebees, this study and Sadd et al. (2015). Estimates in italics are new to this study.

As expected, most of the CO tracts are >100 kb (∼91.4%) or 500 kb (∼87.6%) (supplementary table S3, Supplementary Material online). Restricting analysis to larger spans reduces the estimate to 8.0 cM/Mb for spans >100 kb and 7.7 cM/Mb for spans >500 kb. This suggests that differences between our estimate and the prior one (Stolle et al. 2011) may be marker density, our higher density permitting higher resolution and thus discovery of smaller crossover events. To compare the recombination rate with the previous study (Stolle et al. 2011), we randomly picked 516 markers in our study and calculated the genetic length for each sample. In 1,000 simulations, an average genetic length of 1,840 cM (standard deviation= 419) is observed, which is not significantly different from the previous estimate (1,902 cM before correction). We thus surmise that the dominant reason the small discrepancy between the prior (∼7.5 cM/Mb) and new (8.7 cM/Mb) estimate is the somewhat greater density of markers in our dataset, giving a better ability to detect smaller recombination events. Note too then when a lower density of markers is used in honeybees (either by considering events longer than 100 kb or by simulation of a reduced panel of markers), the estimate for honeybee is still much higher than observed in bumblebees at ∼20 cM/Mb (Liu et al. 2015).

Similar to previous studies in several other species (for review see de Massy 2013), the distribution of COs is highly uneven along the chromosomes. The recombination rate varies between 0 and 62.5 cM/Mb when measured in nonoverlapping 500 kb windows across chromosomes (supplementary fig. S3, Supplementary Material online). A total of nine CO hotspots locating at ∼4.5 Mb regions were identified (supplementary fig. S3, Supplementary Material online). To compare the CO hotspots in bumblebee with its relative the honeybee, we aligned the bumblebee genome along with honeybee genome (supplementary fig. S4, Supplementary Material online), and determined whether the hotspots in bumblebee overlap with those in honeybee. Using the same methods, 24 CO hotspots (combined 5.8 Mb) were detected in honeybee. No overlapping region between them can be found, suggesting plastic evolution of hotspots. On a more gross level, there is resemblance as, similar to the results from the honeybee, the numbers of COs per chromosome is positively related with chromosome length (*r* = 0.66, *P* = 0.003, supplementary fig. S5*A*, Supplementary Material online), and the per base rate of CO (cM/Mb) is not significantly related with chromosome length (*r* = −0.43, *P* = 0.08, supplementary fig. S5*B*, Supplementary Material online).

### High Recombination Rates Are Associated with High GC Content and Biased Gene Conversion

**Fig. 2:**
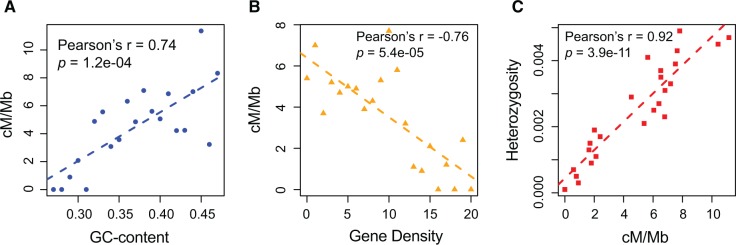
Association between recombination rate and GC-content, heterozygosity and gene density. (*A*) Plot of GC-content against recombination rate. The genome is divided into 100-kb nonoverlapping windows, these windows are sorted and binned by GC-content, then the recombination rate for each bin is calculated. (*B*) Plot of gene density against recombination rate. The genome is divided into 100-kb nonoverlapping windows, these windows are sorted and binned by number of genes within, then the recombination rate for each bin is calculated. (*C*) Plot of nucleotide diversity between the two haplotypes against recombination rate. The genome is divided into 100-kb nonoverlapping windows, these windows are sorted and binned by diversity, then the recombination rate for each bin is calculated.

An association between the recombination rate and GC content is widely reported across eukaryotes (Fullerton et al. 2001; Pessia et al. 2012) and considered a likely consequence of biased gene conversion (Eyrewalker 1993; Birdsell 2002; Duret and Galtier 2009), potentially associated with crossover (Lesecque et al. 2013) or noncrossover (Williams et al. 2015) events. The rate of recombination in bumblebees is indeed highly associated with GC-content (merge method: *r* = 0.74, *P* = 1.2e-04, fig. 2A) and seen in raw window analysis at all blocks sizes (range 10–500 kb; supplementary table S4, Supplementary Material online).

We find evidence supportive of biased gene conversion associated with NCO events. We identified 22 NCOs in all of these 32 drones, ∼0.69 on an average in each drone. These events combined converted 44 SNPs. The direction of conversion is significantly biased towards GC (*P* < 0.001 with 10,000 randomizations): the number of A/T-> G/C conversions to G/C-> A/T conversions is 23:13. Stronger biased gene conversion has been indirectly inferred in honeybees (Wallberg et al. 2015).

In addition to NCOs, we also identified 3 CO-associated gene conversions. In the previous honeybee study (Liu et al. 2015), we identified 82 COs and 5 NCOs per drone, thus hypothesized that the elevated COs may be associated with a reduction in NCOs. In this scenario, for a given species, if the rate of CO is reduced, an elevated rate of NCO could be observed. However, in bumblebee, both the rates of CO and NCO are all much lower than honeybee, indicating there may not be a negative relation between the rates of CO and NCO.

We note one peculiarity, this being that despite a much higher recombination rate (and NCO rate) (table 1), honeybee has a lower average GC content than bumblebee. Thus, whilst the biased gene conversion hypothesis makes robust sense of the trends seen within a genome it does not well predict between-genome differences, at least in this instance. Nonetheless, these results underpin the need to estimate mutation rates in a manner that minimizes the influence of biased gene conversion on allele frequencies.

### Inter- and Intra-Genomic Trends in Heterozygosity Are Consistent with Hill Robertson Interference

Hill–Robertson interference (Hill and Robertson 1966) proposes that, in regions of low recombination rate, the efficiency of selection acting on two linked loci is considerably reduced. Moreover, domains of low recombination are prone to both hitchhiking and background selection, both of which tend to reduce intra-specific diversity. An association between high recombination rates and high heterozygosity is thus expected both within and between genomes. As then expected, the mean heterozygosity is higher in the more highly recombining honeybee (0.35% in honeybee, 0.26% in the bumblebee: these numbers refer to heterozygosity rates within our samples).

Within the bumblebee genome, as expected from Hill Robertson interference, the rate of recombination is positively correlated with heterozygosity (merge method *r* = 0.92, *P* = 3.9e-11, figure 2*C* and, for raw window analysis supplementary table S4, Supplementary Material online). While this is consistent with expectations, it is, however, desirable to control for covariance with gene density effects (McGaugh et al. 2012) as a high density may enforce a low diversity owing to strong selection on deleterious mutations, which can have consequences also for linked neutral sites. In mammalian genomes gene density is highest in GC rich parts of the genome (Mouchiroud et al. 1991; Duret et al. 1995) and hence expected to be associated with high recombination rates. By contrast, there is no correlation between coding sequence density and recombination rate in *Drosophila* (Spearman rho = 0.04, *P *=* *0.68, data from (McGaugh et al. 2012), courtesy of Mohamed Noor). In the bumblebee we observe the opposite pattern to that in mammals (merge method *r* = −0.76, *P* = 5.4e-05, fig. 2B) with high recombination associated with low gene density and low coding sequence density (supplementary table S4, Supplementary Material online). While the trend is seen in the raw window analysis, both applying gene density and coding sequence density as measures (supplementary table S4, Supplementary Material online), the negative correlation turns nonsignificant for the largest window size. The correlation between heterozygosity and recombination in bumblebees is stronger when controlling for gene density or coding sequence density (supplementary table S5, Supplementary Material online). We confirm therefore that recombination is correlated with heterozygosity.

### Honeybees and Bumblebees Have Highly Similar Mutation Rates

The highly covered and accurate sequences in this study provide a unique opportunity to screen germline *de novo* mutations in the bumblebee. First, the specific SNPs were identified in each drone as candidate mutations (supplementary fig. S1, Supplementary Material online), then these SNPs were screened by a series of criteria to exclude false positives (Methods for details). Finally, a total of 23 nucleotide substitutions in the 32 drone genomes are identified as *de novo* mutations (table 2). The combined length of assembled chromosomes is 216.85 Mb, and the callable sites in each drone is 200.82 Mb on an average (supplementary table S1, Supplementary Material online), so the mutation rate is 23/(32 × 200.82 Mb) = 3.58 × 10^−^^9^ base mutations per generation per site per haploid genome. This is highly similar to the mutation rate of honeybee (3.4 × 10^−^^9^ base mutations per generation per site per haploid genome; table 1).

**Table 2:** List of Mutations Identified in Drones.

Number	Sample	Position	Type
1	I-D6	LG1:12190579	G->A
2	I-D9	LG2:6364117	G->A
3	I-D12	LG2:3927622	C->T
4	I-D20	LG3:1376136	G->A
5	I-D15	LG3:5786680	T->C
6	I-D22	LD3:14425758	G->A
7	I-D1	LG6:10066783	C->T
8	I-D18	LG8:4772520	C->T
9	I-D21	LG8:4887207	C->T
10	I-D4	LG8:1918198	C->T
11	I-D15	LG9:4539315	G->A
12	I-D20	LG9:10120753	A->G
13	I-D1	LG12:6547520	G->A
14	I-D1	LG12:317275	T->C
15	I-D13	LG13:4778391	G->A
16	I-D19	LG13:5813863	G->A
17	I-D15	LG15:8684993	C->T
18	II-D21	LG11:10939409	C->T
19	II-D4	LG5:3035192	C->T
20	II-D4	LG5:5897739	G->A
21	II-D16	LG1:7973774	G->A
22	II-D17	LG1:204068	G->A
23	II-D16	LG7:12154508	C->T
24	I-D6	LG1:7408143	“GATTCCGATTCGGATTCC” deletion
25	II-D14	LG14:6588726	“C” deletion

To estimate confidence intervals we assume that the mutation count is approximately Poisson distributed. With 23 mutations the 95% confidence intervals are 14.6 and 34.5. With 32 individuals and a total of 6426135514 callable sites across the 32 individuals, this equates to bounds of 2.38 × 10^−^^9^ (14.6/6426135514) and 5.37 × 10^−^^9^ (34.5/6426135514). These confidence intervals rely on the underlying distribution as being Poisson. We simulate a random allocation of 23 mutations between 32 individuals and compare the observed and randomized variance of the mutation count between individuals. Note that in a Poisson process we expect the variance to equal the mean. As in the simulations the mean is fixed to the observed mean (23/32 = 0.72), we need only ask whether the variance of the observations is equal to the variance of the simulants. We thus ask in how many simulants the observed variance is as great or greater than the randomized variance. We see no significant difference (*P *=* *0.75, Monte Carlo randomization, 100,000 randomizations) between random (mean variance = 0.719, 95% limits 0.40, 1.11) and observed (variance = 0.789) and thus fail to reject the null hypothesis of no over- or under-dispersion (i.e., Variance/Mean = 1), the hallmark of a Poisson process.

The above mutation rate estimation suggests that the difference between the two bee species is not significant. To determine this more rigorously we started by estimating the per genome per bp mutation rate for each individual in this and our prior analysis (43 in honeybee and 32 in bumblebees). We took the mean of these estimates for each species and considered the parameter delta, this being the difference in mean per individual per genome per bp mutation rate. We then randomly drew 43 and 32 individuals from the set of 75 individuals and calculated the mean mutation rate for each randomized partition, then considered delta for the pair of randomized partitions. We repeat the randomization asking how often (count =*n*) the randomized delta is as great or greater than the observed delta. We find that out of 10,000 simulations, randomized delta is as great or greater than observed delta for 8,245 times, so *P *=* *0.8245.

In addition to SNP mutations, we also identified 2 indel mutation in the 32 drones, a 1-bp deletion and an 18-bp deletion (table 2). The rate of indel mutation is 3.1 ×10^−^^10^ events per generation per site per haploid genome (95% confidence intervals = 3.8 ×10^−^^11^ ∼ 1.1 × 10^−^^9^), which is lower but not significantly different from the indel mutation rate in honey bee (1.0 × 10^−^^9^, 95% confidence intervals = 4.4 ×10^−^^10^ ∼ 2.0 × 10^−^^9^). The indel to point mutation ratio (1:11.5) is rather lower than some prior estimates (Ossowski et al. 2010; Jiang et al. 2014; Yang et al. 2015), which are typically in the range 3.11–5.8. However, compared with our data in *Arabidopsis* (67 indels and 237 point mutations), our bumblebee ratio is not significantly different (chi-squared =2.74, *P* = 0.097).

### Mutation Is GC to AT Biased

As seems almost universal (Hershberg and Petrov 2010; Hildebrand et al. 2010; Lynch 2010; Ossowski et al. 2010), the spectrum of mutations is significantly biased favoring G:C-> A:T transitions. All of the 23 mutations are transitions, and 20 of them are G:C-> A:T transitions, whereas only 3 of them are A:T-> G:C transitions (table 2). Note that, in addition, these are raw counts uncorrected for underlying GC content. At the limit, were the genome nearly all GC then naturally there would be very few AT->GC in absolute terms. However, the genome is AT biased in underlying content (GC%=37.5%), making the excess of GC->AT all the more striking. Correcting for base content we estimate a 53.3:4.8 ratio of GC->AT per GC compared with AT->GC per AT, this trend is similar to the estimates in honeybee (55.9:6.1 from direct parent-offspring sequencing; Yang et al. 2015, and 60:10 inferred from population level analysis; Wallberg et al. 2015).

### Evidence for Recombination-Associated Mutation but the Effect Is Very Weak

Prior evidence from honeybees suggested a small but significant excess of mutations in the near vicinity of recombination break points (Yang et al. 2015). We observe 1 of the 23 mutations in proximity (< 1 kb) of a crossover event in bumblebees. The distance from the mutation to crossover is only 146 bp. To determine whether this is more than expected by chance, we randomly selected 23 sites in the genome as pseudo-mutational sites, and asked how many of these 23 are within 1 kb of a recombination breakpoint. From repeat of the simulation we find one or more mutations in vicinity in the set of 23 approximately in 73 out of 10,000 simulations, *P*= 0.0074. While one mutation is no grounds for declaring strong confidence in the theory (Magni and von Borstel 1962; Magni 1964), this observation added to the significant excess also seen in honeybees (Yang et al. 2015), suggests that possibly ∼5% of *de novo* mutations are in close proximity to recombination events (2 of 35 were in proximity in honeybee, 1 of 23 in bumblebees = 3/55 = 0.052).

The altered recombination rate seems to be a very minor player in determining between-species mutation rates. The rate at which COs are associated with mutations is 1/606 = 0.0017 in bumblebee and 2/3,505 = 0.00057 in honeybee. Bumblebees have 19 COs per drone, honeybees have 82 COs per drone. So the number of mutations introduced by CO per drone is: 19 × 0.0017= 0.03 in bumblebee and 82 × 0.00057 = 0.05 in honeybee. Thus the effect, while significant, amounts to only 0.02 more mutations per drone in honeybees (i.e., 1.1 × 10^−^^10^ base mutations per generation per site per haploid genome) owing to their increased recombination rate. Thus, while we can detect a weak signal of recombination-associated mutations, it is a minor contributor to between-species differences.

### No Evidence for Between-Individual Variation in the Mutation Rate

Variation within a species between individuals in the mutation rate has been described (Baer et al. 2005; Schrider et al. 2013; Ness et al. 2015), with some intraspecifc variation being associated with fitness differences with low fitness individuals having higher rates (for review Baer 2008). Between the progeny in colony I and colony II we see no evidence for a difference in mutation rate (colony I has 22 individuals and 17 mutations with 15.81 expected, colony II has 10 individuals and 6 mutations and 7.19 expected; chi-squared = 0.25, d.f. =1, *P *≫* *0.05).

### The Per Genome Mutation Rate Is Less than One

Estimation of the mutation rate is also of relevance to models for the evolution of sex. The mutational deterministic model, for example, predicts that obligately sexual taxa (such as honeybees and bumblebees) must have more than one deleterious mutation per genome per generation (Kondrashov 1988). After making allowance for the unsequenced part of the genome, both bees have on an average nearly one new mutation per genome per generation (estimate ∼0.98 in bumblebees, 0.8 in honey bees). To estimate the rate of deleterious mutations these numbers need to be scaled by the proportion of mutations that are deleterious. This we have not attempted to estimate, but we note that as the per generation per genome rates are not in excess of unity, it is unlikely that these species have an adequate input of deleterious mutations to satisfy the mutational deterministic model.

## Discussion

Here we present the fourth direct estimate of the mutation rate for an insect species. Our estimate for the bumblebee is highly similar to that of the honeybee at ∼3.5 × 10^−^^9^ per base pair per genome. We could confirm that the recombination rate of the bumblebee is considerably lower than that of the honeybees, the precise magnitude of difference being dependent on the marker density (7.5: 20 cM/Mb at low density, 8.7: 37 cM/Mb at higher densities). Intra-genomically, the expected correlates to higher recombination rates are seen, notably higher heterozygosity and higher GC-content. The GC-recombination correlation is typically interpreted as owing to GC biased gene conversion (Eyrewalker 1993; Birdsell 2002; Duret and Galtier 2009). We find evidence that biased gene conversion operates in bumblebees. Inter-genomically, the issue is not as straightforward. While heterozygosity is higher in the more recombining honeybee, GC content is lower. The mutational profile in the bumblebee is very strongly skewed in favor of AT (53.3:4.8 ratio of GC->AT per GC compared with AT->GC per AT). As this trend is similar to that in honeybee (55.9:6.1) a mutational explanation appears unlikely to explain bumblebees higher GC content. A possible explanation for this apparently contradictory observation is that selection is also stronger when the recombination rate is higher, so deleterious AT->GC SNPs may be opposed more efficiently in honeybees when they are not favored by biased gene conversion.

### No Evidence That Differences in Recombination Rate Majorly Effect Mutation Rates

We detected a weak effect whereby increased recombination might have directly led to more mutations in bees, as we find further evidence that recombination and mutation are coupled in this taxa. This effect is however so weak that a trebling in the recombination rate may have led to only a very slight increment (0.02 new mutations per drone) in the total number of new mutations (a difference that is far beyond our resolution to discern). To more fully resolve this weak effect will require at least an order of magnitude more sequencing effort. Nonetheless, given the abundant recombination in bees, they would be good material for such analysis.

The lack of a lower mutation rate in the species with the lower recombination rate has, in principle, bearing on other theories of mutation generation. Indeed, higher recombination rates could have lead to higher mutation rates for reasons other than recombination being directly mutagenic. A potentially viable alternative model supposes that (1) increased recombination permits higher heterozygosity via reduced Hill–Robertson inference (Hill and Robertson 1966) and (2) heterozygosity is mutagenic (Duncan 1915; Emerson 1929; Demerec 1932; Timofeeff-Ressovsky 1932; Amos 2010b). As we find, a relationship between heterozygosity and recombination is commonly reported (Smukowski and Noor 2011; Cutter and Payseur 2013) and can be distinguished from any possible effect of recombination being mutagenic (McGaugh et al. 2012). By contrast, the conjecture that heterozygosity and mutation are directly coupled processes, although an ancient hypothesis (Duncan 1915; Emerson 1929; Demerec 1932; Timofeeff-Ressovsky 1932), is far from demonstrated.

While increased mutation rates in some between-species and between-population hybrids is well described (Belgovsky 1937; Sturtevant 1939; Woodruff et al. 1979; Simmons et al. 1980; Thompson and Woodruff 1980; Bashir et al. 2014), this may reflect epistatic effects (Sturtevant 1939; Lynch 2015), not direct effects of heterozygosity per se. That the raised mutation rates in hybrids are seen in haploid parts of the genome (Simmons et al. 1980; Thompson and Woodruff 1980) (i.e., the X), that the effects are commonly reported to be dependent on the direction of the cross (Sturtevant 1939; Simmons et al. 1980; Bashir et al. 2014), and that the effects are independent of genetic distance (Bashir et al. 2014), all argue against heterozygosity as directly causative. Indeed, outside of the context of hybrids, experimental tests of the hypothesis are largely negative (Demerec 1932; Timofeeff-Ressovsky 1932). In contrast to these experimental manipulations of heterozygosity, the recent evidence advocated (Amos 2010a, 2010b, 2013) as being consistent with the hypothesis is largely correlation-based (for review Amos 2010b) and subject to alternative interpretation. For example, while SNP clustering (see, e.g., Smith and Lercher 2002) has been interpreted (Amos 2010a) as consistent with heterozygosity-induced mutations, many forces affect regional mutation rates, on many different scales (Hodgkinson and Eyre-Walker 2011; Schuster-Bockler and Lehner 2012; Makova and Hardison 2015), and provide parsimonious alternative explanation, under a neutral null model. Similarly, while the correlation between substitution rate and recombination (Lercher and Hurst 2002)/heterozygosity, is presented as evidence for the heterozygosity-mutation hypothesis (Amos 2010b), it is parsimoniously explained by biased gene conversion (Duret and Arndt 2008). The observation of higher divergence from chimp in more heterozygous human populations (Africans vs. non-Africans) (Amos 2013), that is seen only on autosomes and not on X or Y of African populations, may well have a similar explanation, as biased gene conversion has modulated branch lengths between human populations (Lachance and Tishkoff 2014). A recent observation of increased mutation rates in genomic sub-compartments made to be heterozygous (Yang et al. 2015) is the most suggestive evidence that we are aware of, at least as regards point mutations, that mutation and heterozygosity can sometimes be coupled.

Unfortunately, this present analysis has little to add to this debate. As expected, heterozygosity is higher in the species with more crossing over and, in the bumblebee genome, in domains of high crossover rates. Despite the lower recombination rate and lower heterozygosity, we see no evidence that bumblebee has a lower per bp per haploid genome mutation rate. *Prima facie* then we fail to support the heterozygosity-mutation hypothesis as a mode of explaining between-species differences in the mutation rate. However, this comes with the major caveat that the bounds of our 95% errors are quite wide. Indeed, there is only a 35% difference in heterozygosity levels between the two species in our samples and we cannot reject the hypothesis that the bumblebee mutation rate is approximately one-third lower than that observed in honeybees. If the mutation rates were in proportion to the heterozygosity rates then we predict a rate of 2.5×10^−^^9^ for the bumblebee, given a 3.4×10^−^^9^ in the honeybee, this being slightly above our lower confidence bound of 2.38×10^−^^9^. Thus we can only conclude that our analysis has failed to provide support for the hypothesis that the honeybee mutation rate has been affected by its higher heterozygosity.

### Is the Mutation Rate in Insects Constant?

Our estimates of the mutation rates for the two bees are also similar to the two other estimates via comparable parent-offspring sequencing in insects, in *Heliconius melpomene* (Keightley et al. 2015) and *Drosophila melanogaster* (Keightley et al. 2014), these being estimated as 2.9 × 10^−^^9^ (with an upper 95% limit of 5.5 × 10^−^^9^) and 2.8 × 10^−^^9^ (with an upper 95% limit of 6.1 × 10^−^^9^), respectively. With the confidence bounds set as they are, we note that we cannot currently exclude the possibility that all insects have the same mutation rate. The similarity in the four rate estimates further supports the view that sociality, although associated with high recombination rates, is not associated with high mutation rates. Indeed the data suggest an approximate constancy of the mutation rate in insects. Given this, the ancestral mutation rate for the Corbiculates is probably of the order of 2.8–3.5 mutations per base pair per generation per haploid genome.

Against the thesis of approximate constancy of the mutation rate within insects, we note that a further relatively direct estimate from *Drosophila* (Sharp and Agrawal 2016), from mutation accumulation lines, estimates the rate to be 3 times higher than the parent-offspring sequencing estimate at 6.03 × 10^−^^9^. For similar slightly higher estimates see also (Haag-Liautard et al. 2007; Schrider et al. 2013). As the differences between *Drosophila* estimates could reflect interesting biology or technical method specifications, it is unclear how to interpret these numbers. Further, the hypothesis of approximate constancy of the mutation rate ignores the possibility of between-lineage intra-species differences (Baer et al. 2005; Schrider et al. 2013; Ness et al. 2015). While we detected no evidence for such an effect our analysis had very limited power as we only have two lines.

## Supplementary Material

Supplementary figures S1–S5 and tables S1–S5 are available at *Molecular Biology and Evolution* online.

## Author Contributions

S.Y., L.D.H., and D.T. conceived the study and designed the experiments. H.L., Y.J., and X.S. performed the experiments. L.D.H., S.Y., and H.L. analyzed the data and wrote the article. All authors have read and approved the article for publication.

## Supplementary Material

Supplementary Data
