# Supplementary material for: Direct Determination of the Mutation Rate in the Bumblebee Reveals Evidence for Weak Recombination-Associated Mutation and an Approximate Rate Constancy in Insects
Source: Mol Biol Evol. 2016 Oct 20;34(1):119–30. doi: 10.1093/molbev/msw226 (PMC5854123; doi:10.1093/molbev/msw226)

**Figure S1. Schematic diagram of identification of mutation candidates.** Genotypes of 22 drones on part of chromosome 1 in shown in figure below, the two different haplotypes are shown in blue and red. If a specific SNP is genotyped in a drone that is different from all the other drones, this specific SNP is identified as a mutation candidate. Illustrated in this figure, a mutation candidate identified in sample D6 is highlighted by green shade.


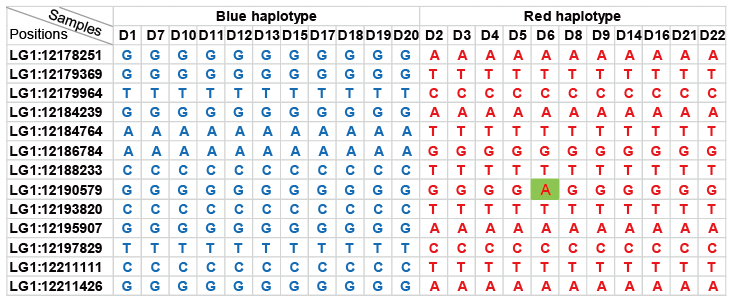


**Figure S2. Schematic diagram of the process of haplotype phasing along the chromosome.** Genotypes of 22 drones on part of chromosome 10. A crossover event can be identified in sample D2.


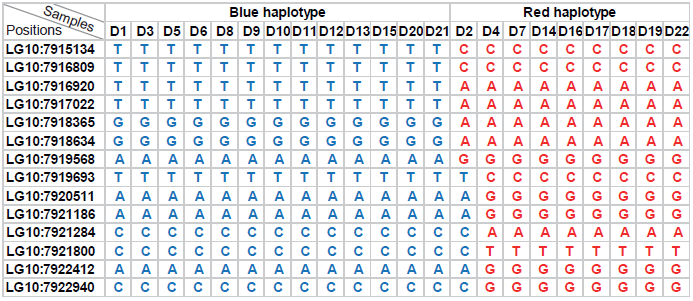


**Figure S3. Recombination rate and marker density variation along chromosomes 1~18.** The genome is divided into 500 kb non-overlapping windows, recombination rate and marker density for each window is calculated. Recombination rate is represented by the blue line and the vertical bar on the left, marker density is represented by the red dotted line and the vertical bar on the right. Recombination rate above the yellow dotted line is CO hotspot for *p*<0.01.


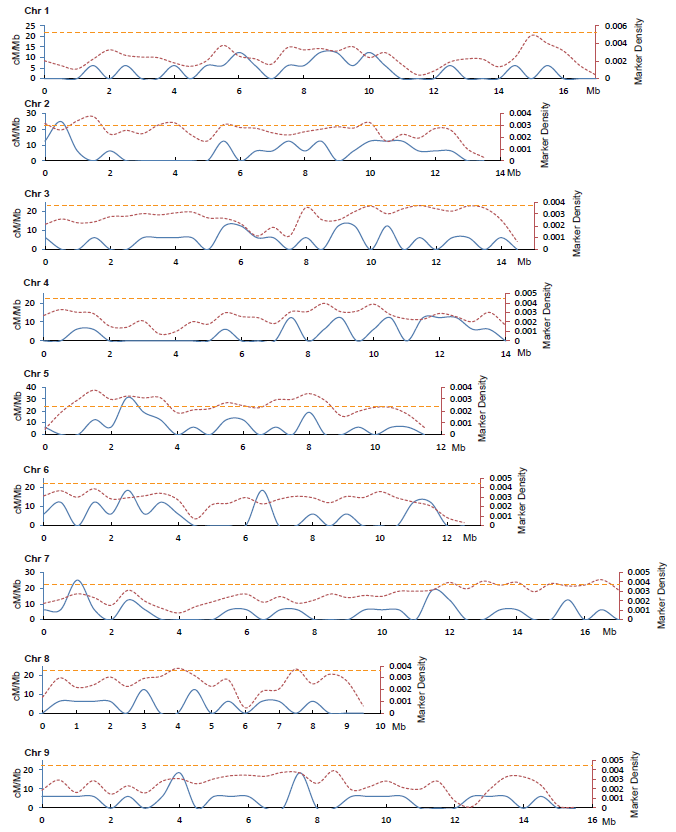


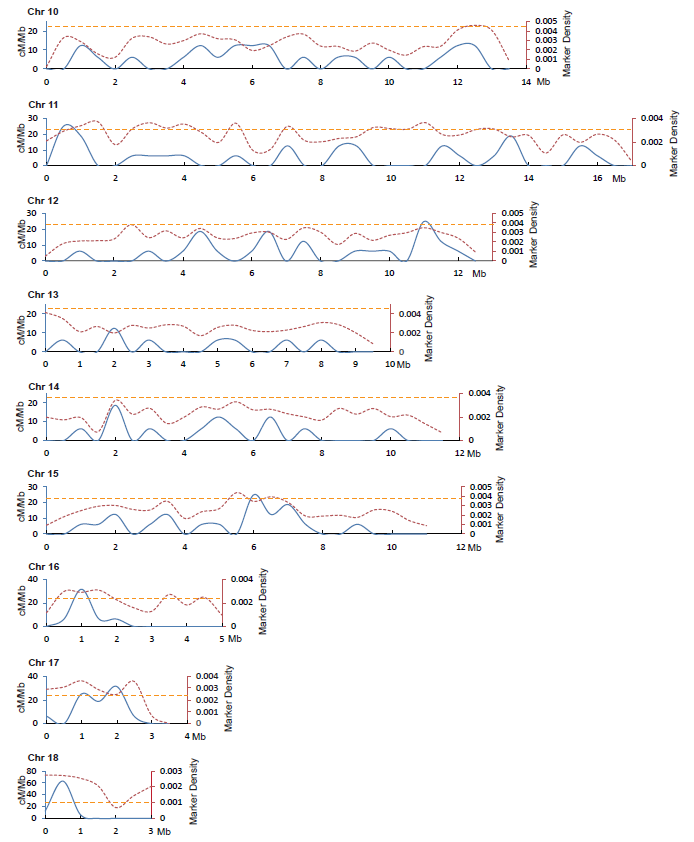


**Figure S4. Genome alignment between bumblebee and honey bee.** Bumblebee chromosomes are represented by blue bars on the left while honey bee by yellow bars on the right. Red windows on chromosomes represent CO hotspots. The bumblebee genome is dissected into 1 kb non-overlapping sequences, and blast against the honey bee genome, each best hit is linked by a green or red line. Sequences in bumblebee hotspots are linked by red. For 89% of these sequences, homologous sequence can be found with identify ≥70% and alignment length ≥100bp.


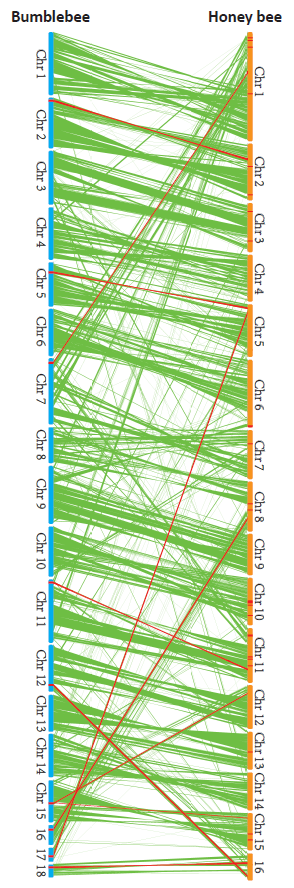


**Figure S5. Plot of chromosome length against average number of crossovers (A.) or crossover rate (B.) on each chromosome.**


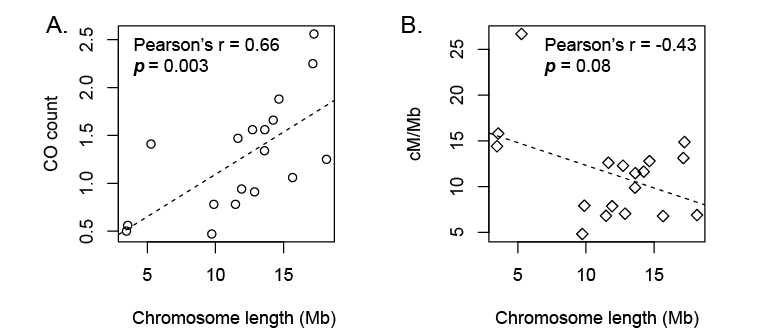

Supplement: Supplementary Data [file msw226_suppl.zip › Supplementary_Figures.docx]
