# Supplementary material for: Direct Determination of the Mutation Rate in the Bumblebee Reveals Evidence for Weak Recombination-Associated Mutation and an Approximate Rate Constancy in Insects
Source: Mol Biol Evol. 2016 Oct 20;34(1):119–30. doi: 10.1093/molbev/msw226 (PMC5854123; doi:10.1093/molbev/msw226)
Supplement: Supplementary Data [file msw226_suppl.zip › TablesS.28082016.docx]

**Table S1. Summary statistics for sequencing data.**

| Colony I | | | |
| --- | --- | --- | --- |
| Sample | Genome Coverage | Mean Depth | Callable sites* |
| I-D1 | 98% | 28.13 | 201,273,205 |
| I-D2 | 99% | 26.22 | 201,870,269 |
| I-D3 | 96% | 21.97 | 195,452,029 |
| I-D4 | 98% | 31.47 | 201,121,239 |
| I-D5 | 99% | 29.05 | 201,950,511 |
| I-D6 | 99% | 32.18 | 203,272,529 |
| I-D7 | 99% | 24.32 | 200,128,439 |
| I-D8 | 97% | 22.00 | 196,691,902 |
| I-D9 | 99% | 37.84 | 204,668,268 |
| I-D10 | 98% | 22.52 | 201,281,475 |
| I-D11 | 98% | 22.47 | 201,319,644 |
| I-D12 | 99% | 22.34 | 202,454,543 |
| I-D13 | 99% | 26.57 | 202,786,909 |
| I-D14 | 98% | 28.34 | 200,487,213 |
| I-D15 | 99% | 26.97 | 202,189,312 |
| I-D16 | 98% | 28.67 | 201,113,114 |
| I-D17 | 99% | 25.48 | 202,037,304 |
| I-D18 | 99% | 28.36 | 204,361,412 |
| I-D19 | 99% | 36.87 | 203,185,488 |
| I-D20 | 99% | 26.39 | 202,075,346 |
| I-D21 | 99% | 25.67 | 202,329,544 |
| I-D22 | 93% | 24.15 | 187,109,287 |
| Colony II | | | |
| Sample | Genome Coverage | Mean Depth | Callable sites* |
| II-D2 | 99% | 29.27 | 202,678,063 |
| II-D4 | 99% | 25.99 | 200,609,920 |
| II-D8 | 99% | 23.46 | 200,609,910 |
| II-D9 | 98% | 24.28 | 200,610,132 |
| II-D12 | 99% | 25.24 | 200,609,688 |
| II-D14 | 99% | 23.23 | 198,541,776 |
| II-D16 | 99% | 24.86 | 200,610,031 |
| II-D17 | 99% | 24.18 | 198,541,776 |
| II-D21 | 99% | 23.80 | 202,678,054 |
| II-D27 | 99% | 21.36 | 200,609,809 |

*Callable sites is defined as read depth ≥5 and mapping quality ≥20 and no ambiguous bases in reference genome.

**Table S2. List of crossover events identified in this study.** The first column is sample, the second and third columns are the nearest markers around crossover breakpoint. Crossover takes place between these two markers.

| Sample | Breakpoint markers | |
| --- | --- | --- |
|  | **Start** | **End** |
| I-D1 | LG10:11339577 | LG10:11399306 |
| I-D1 | LG10:12361566 | LG10:12361979 |
| I-D1 | LG10:8878269 | LG10:8886519 |
| I-D1 | LG11:11744226 | LG11:11744789 |
| I-D1 | LG12:6546989 | LG12:6547374 |
| I-D1 | LG15:9416170 | LG15:9416840 |
| I-D1 | LG17:1429029 | LG17:1429077 |
| I-D1 | LG18:825724 | LG18:826756 |
| I-D1 | LG2:12190784 | LG2:12191131 |
| I-D1 | LG2:5389498 | LG2:5440784 |
| I-D1 | LG3:10180691 | LG3:10232580 |
| I-D1 | LG3:6633229 | LG3:6685979 |
| I-D1 | LG4:3178502 | LG4:3242555 |
| I-D1 | LG4:813707 | LG4:869821 |
| I-D1 | LG7:13409566 | LG7:13414329 |
| I-D1 | LG7:1722667 | LG7:1726535 |
| I-D1 | LG7:5260632 | LG7:5264492 |
| I-D1 | LG8:3218592 | LG8:3219507 |
| I-D2 | LG1:1652080 | LG1:1652926 |
| I-D2 | LG10:5046055 | LG10:5047013 |
| I-D2 | LG10:7919568 | LG10:7919693 |
| I-D2 | LG11:17156341 | LG11:17176778 |
| I-D2 | LG11:9155829 | LG11:9156281 |
| I-D2 | LG11:930789 | LG11:932090 |
| I-D2 | LG12:1314111 | LG12:1317849 |
| I-D2 | LG12:7640710 | LG12:7642732 |
| I-D2 | LG13:1216427 | LG13:1269379 |
| I-D2 | LG13:800319 | LG13:800880 |
| I-D2 | LG15:6558825 | LG15:6559692 |
| I-D2 | LG16:2310752 | LG16:2314436 |
| I-D2 | LG18:242233 | LG18:242628 |
| I-D2 | LG2:11833033 | LG2:11834100 |
| I-D2 | LG3:7031899 | LG3:7033737 |
| I-D2 | LG4:3178502 | LG4:3242555 |
| I-D2 | LG5:2844333 | LG5:2844572 |
| I-D2 | LG7:1077075 | LG7:1080927 |
| I-D2 | LG7:11544571 | LG7:11545618 |
| I-D2 | LG9:10270450 | LG9:10270879 |
| I-D3 | LG1:7984962 | LG1:7986627 |
| I-D3 | LG10:11339577 | LG10:11399306 |
| I-D3 | LG10:4544922 | LG10:4545201 |
| I-D3 | LG11:11696814 | LG11:11700137 |
| I-D3 | LG11:17156341 | LG11:17176778 |
| I-D3 | LG11:2827193 | LG11:2827865 |
| I-D3 | LG13:5167147 | LG13:5169557 |
| I-D3 | LG14:8755430 | LG14:8758440 |
| I-D3 | LG16:2444409 | LG16:2495812 |
| I-D3 | LG18:361036 | LG18:363575 |
| I-D3 | LG2:6528676 | LG2:6530661 |
| I-D3 | LG3:7155425 | LG3:7859148 |
| I-D3 | LG4:3178502 | LG4:3242555 |
| I-D3 | LG5:6385688 | LG5:6386335 |
| I-D3 | LG6:507139 | LG6:507432 |
| I-D3 | LG6:6862403 | LG6:6863298 |
| I-D3 | LG7:10998940 | LG7:10999974 |
| I-D3 | LG8:1794417 | LG8:1795005 |
| I-D4 | LG1:8519669 | LG1:8529565 |
| I-D4 | LG10:78978 | LG10:198039 |
| I-D4 | LG10:8715441 | LG10:8720797 |
| I-D4 | LG11:13989843 | LG11:13990369 |
| I-D4 | LG11:14614588 | LG11:14738808 |
| I-D4 | LG11:15624684 | LG11:15625990 |
| I-D4 | LG17:2210507 | LG17:2210986 |
| I-D4 | LG2:160430 | LG2:162759 |
| I-D4 | LG3:7155425 | LG3:7859148 |
| I-D4 | LG4:8604311 | LG4:8614649 |
| I-D4 | LG7:10932024 | LG7:10934902 |
| I-D4 | LG9:2337663 | LG9:2460850 |
| I-D4 | LG9:351090 | LG9:351790 |
| I-D5 | LG10:6336139 | LG10:6336942 |
| I-D5 | LG11:1300653 | LG11:1300978 |
| I-D5 | LG11:17156341 | LG11:17176778 |
| I-D5 | LG13:1216427 | LG13:1269379 |
| I-D5 | LG13:3225611 | LG13:3226358 |
| I-D5 | LG13:4539690 | LG13:4602911 |
| I-D5 | LG14:4869348 | LG14:4869435 |
| I-D5 | LG15:1077 | LG15:59251 |
| I-D5 | LG15:313017 | LG15:321577 |
| I-D5 | LG16:2444409 | LG16:2495812 |
| I-D5 | LG17:1918300 | LG17:1919683 |
| I-D5 | LG2:10404449 | LG2:10405064 |
| I-D5 | LG2:834036 | LG2:834983 |
| I-D5 | LG3:6633229 | LG3:6685979 |
| I-D5 | LG4:12056444 | LG4:12057254 |
| I-D5 | LG4:813707 | LG4:869821 |
| I-D5 | LG5:2094836 | LG5:2097149 |
| I-D5 | LG5:8326517 | LG5:8329526 |
| I-D5 | LG6:9595251 | LG6:9597955 |
| I-D5 | LG7:9768967 | LG7:9769457 |
| I-D5 | LG8:4607586 | LG8:4607615 |
| I-D5 | LG9:9250853 | LG9:9251036 |
| I-D6 | LG10:11339577 | LG10:11399306 |
| I-D6 | LG10:12931257 | LG10:12931603 |
| I-D6 | LG10:198039 | LG10:445709 |
| I-D6 | LG10:9362965 | LG10:9363846 |
| I-D6 | LG11:11973969 | LG11:11976655 |
| I-D6 | LG11:17156341 | LG11:17176778 |
| I-D6 | LG11:991469 | LG11:991504 |
| I-D6 | LG16:2444409 | LG16:2495812 |
| I-D6 | LG18:948136 | LG18:949302 |
| I-D6 | LG2:3287073 | LG2:3294309 |
| I-D6 | LG2:7547384 | LG2:7547850 |
| I-D6 | LG3:13846699 | LG3:13849296 |
| I-D6 | LG3:6633229 | LG3:6685979 |
| I-D6 | LG4:3178502 | LG4:3242555 |
| I-D6 | LG4:813707 | LG4:869821 |
| I-D6 | LG6:10623264 | LG6:10626028 |
| I-D6 | LG6:669808 | LG6:670479 |
| I-D7 | LG10:4959011 | LG10:4962140 |
| I-D7 | LG12:8337654 | LG12:8340361 |
| I-D7 | LG13:1216427 | LG13:1269379 |
| I-D7 | LG13:2165861 | LG13:2167349 |
| I-D7 | LG13:4604482 | LG13:4658589 |
| I-D7 | LG15:1044427 | LG15:1046131 |
| I-D7 | LG15:1459404 | LG15:1511616 |
| I-D7 | LG15:7150669 | LG15:7150962 |
| I-D7 | LG15:838371 | LG15:890963 |
| I-D7 | LG16:2444409 | LG16:2495812 |
| I-D7 | LG17:2142769 | LG17:2143528 |
| I-D7 | LG18:598365 | LG18:599613 |
| I-D7 | LG2:10288817 | LG2:10290582 |
| I-D7 | LG2:7012054 | LG2:7013630 |
| I-D7 | LG3:7155425 | LG3:7859148 |
| I-D7 | LG3:9900192 | LG3:9902812 |
| I-D7 | LG4:12882724 | LG4:12883434 |
| I-D7 | LG4:3178502 | LG4:3242555 |
| I-D7 | LG4:813707 | LG4:869821 |
| I-D7 | LG4:8579120 | LG4:8584060 |
| I-D7 | LG5:8208265 | LG5:8209339 |
| I-D7 | LG7:1142176 | LG7:1144915 |
| I-D7 | LG8:5333649 | LG8:5335729 |
| I-D7 | LG8:6988124 | LG8:6989498 |
| I-D7 | LG9:13806243 | LG9:13806252 |
| I-D8 | LG1:9519752 | LG1:9520576 |
| I-D8 | LG10:198039 | LG10:445709 |
| I-D8 | LG10:9214270 | LG10:9216826 |
| I-D8 | LG11:7086536 | LG11:7087912 |
| I-D8 | LG14:5429411 | LG14:5430273 |
| I-D8 | LG18:815177 | LG18:815525 |
| I-D8 | LG2:10686648 | LG2:10687060 |
| I-D8 | LG3:1325451 | LG3:1329484 |
| I-D8 | LG3:7155425 | LG3:7859148 |
| I-D8 | LG3:9295263 | LG3:9296986 |
| I-D8 | LG4:3178502 | LG4:3242555 |
| I-D8 | LG4:813707 | LG4:869821 |
| I-D8 | LG5:3996797 | LG5:3998441 |
| I-D8 | LG6:3988043 | LG6:3989302 |
| I-D8 | LG7:2916591 | LG7:2918319 |
| I-D8 | LG9:2886384 | LG9:2886793 |
| I-D9 | LG1:1021647 | LG1:1140223 |
| I-D9 | LG1:75333 | LG1:77349 |
| I-D9 | LG10:11339577 | LG10:11399306 |
| I-D9 | LG10:12136160 | LG10:12137478 |
| I-D9 | LG10:5956369 | LG10:5967003 |
| I-D9 | LG11:14614588 | LG11:14738808 |
| I-D9 | LG11:16078262 | LG11:16079101 |
| I-D9 | LG12:11075559 | LG12:11076760 |
| I-D9 | LG13:5455411 | LG13:5456737 |
| I-D9 | LG15:3335711 | LG15:3335818 |
| I-D9 | LG16:3562473 | LG16:3565409 |
| I-D9 | LG17:2188461 | LG17:2189378 |
| I-D9 | LG2:14298 | LG2:16966 |
| I-D9 | LG3:3740334 | LG3:3742121 |
| I-D9 | LG3:6633229 | LG3:6685979 |
| I-D9 | LG4:3178502 | LG4:3242555 |
| I-D9 | LG5:3414056 | LG5:3415421 |
| I-D9 | LG6:9335101 | LG6:9337716 |
| I-D9 | LG7:13533243 | LG7:13535300 |
| I-D10 | LG1:5518422 | LG1:5569968 |
| I-D10 | LG1:6350162 | LG1:6351912 |
| I-D10 | LG1:7275048 | LG1:7332652 |
| I-D10 | LG10:11339577 | LG10:11399306 |
| I-D10 | LG10:1350715 | LG10:1352061 |
| I-D10 | LG10:6767933 | LG10:6768607 |
| I-D10 | LG11:17156341 | LG11:17176778 |
| I-D10 | LG11:581810 | LG11:583536 |
| I-D10 | LG12:4952230 | LG12:4953720 |
| I-D10 | LG12:9608170 | LG12:9608426 |
| I-D10 | LG13:1216427 | LG13:1269379 |
| I-D10 | LG13:3322669 | LG13:3337448 |
| I-D10 | LG13:4539690 | LG13:4602911 |
| I-D10 | LG15:7553136 | LG15:7554105 |
| I-D10 | LG16:1880714 | LG16:1880994 |
| I-D10 | LG18:673365 | LG18:673857 |
| I-D10 | LG2:1751229 | LG2:1753439 |
| I-D10 | LG2:5575900 | LG2:5577437 |
| I-D10 | LG3:13157340 | LG3:13159004 |
| I-D10 | LG3:5851140 | LG3:5852066 |
| I-D10 | LG3:7155425 | LG3:7859148 |
| I-D10 | LG4:10724602 | LG4:10725724 |
| I-D10 | LG5:7936999 | LG5:7940363 |
| I-D10 | LG6:11641804 | LG6:11642599 |
| I-D10 | LG6:1570271 | LG6:1570499 |
| I-D10 | LG7:1181373 | LG7:1182475 |
| I-D10 | LG7:11893813 | LG7:11895370 |
| I-D10 | LG7:15806733 | LG7:15807941 |
| I-D10 | LG9:10402248 | LG9:10404284 |
| I-D10 | LG9:1300253 | LG9:1301465 |
| I-D10 | LG9:2337663 | LG9:2460850 |
| I-D11 | LG1:2607148 | LG1:2611686 |
| I-D11 | LG10:11339577 | LG10:11399306 |
| I-D11 | LG10:12244613 | LG10:12286017 |
| I-D11 | LG10:4977629 | LG10:4978297 |
| I-D11 | LG11:13881741 | LG11:13882557 |
| I-D11 | LG11:17156341 | LG11:17176778 |
| I-D11 | LG12:11399409 | LG12:11400430 |
| I-D11 | LG12:6640560 | LG12:6642420 |
| I-D11 | LG13:6109540 | LG13:6113786 |
| I-D11 | LG14:4543351 | LG14:4545871 |
| I-D11 | LG3:4878176 | LG3:4881234 |
| I-D11 | LG3:6633229 | LG3:6685979 |
| I-D11 | LG3:9700792 | LG3:9702712 |
| I-D11 | LG4:9534927 | LG4:9539997 |
| I-D11 | LG6:3608268 | LG6:3609715 |
| I-D11 | LG7:1021443 | LG7:1021934 |
| I-D11 | LG7:7478001 | LG7:7479240 |
| I-D11 | LG9:5584242 | LG9:5584705 |
| I-D12 | LG1:8452081 | LG1:8454065 |
| I-D12 | LG11:17156341 | LG11:17176778 |
| I-D12 | LG11:9824618 | LG11:9828865 |
| I-D12 | LG15:6073921 | LG15:6074183 |
| I-D12 | LG16:1400516 | LG16:1402075 |
| I-D12 | LG17:1073858 | LG17:1074816 |
| I-D12 | LG2:9142936 | LG2:9146101 |
| I-D12 | LG3:10664469 | LG3:10665408 |
| I-D12 | LG3:7155425 | LG3:7859148 |
| I-D12 | LG4:12068134 | LG4:12068730 |
| I-D12 | LG7:12255746 | LG7:12255982 |
| I-D12 | LG7:6268043 | LG7:6268520 |
| I-D13 | LG1:5518422 | LG1:5569968 |
| I-D13 | LG1:6192960 | LG1:6193570 |
| I-D13 | LG1:7275048 | LG1:7332652 |
| I-D13 | LG10:1469578 | LG10:1470350 |
| I-D13 | LG11:5807954 | LG11:5808368 |
| I-D13 | LG11:9243604 | LG11:9246464 |
| I-D13 | LG12:11472043 | LG12:11472430 |
| I-D13 | LG16:2444409 | LG16:2495812 |
| I-D13 | LG17:2270909 | LG17:2271863 |
| I-D13 | LG2:11180732 | LG2:11181898 |
| I-D13 | LG3:6633229 | LG3:6685979 |
| I-D13 | LG4:3178502 | LG4:3242555 |
| I-D13 | LG4:813707 | LG4:869821 |
| I-D13 | LG5:5642623 | LG5:5643470 |
| I-D13 | LG6:2860191 | LG6:2860517 |
| I-D13 | LG8:2214350 | LG8:2216157 |
| I-D13 | LG9:11116175 | LG9:11119127 |
| I-D14 | LG1:10414095 | LG1:10414840 |
| I-D14 | LG11:17156341 | LG11:17176778 |
| I-D14 | LG15:6467398 | LG15:6467600 |
| I-D14 | LG16:156173 | LG16:180408 |
| I-D14 | LG17:1830453 | LG17:1835103 |
| I-D14 | LG2:795298 | LG2:795743 |
| I-D14 | LG4:813707 | LG4:869821 |
| I-D14 | LG4:8648099 | LG4:8650834 |
| I-D14 | LG5:1881919 | LG5:1882905 |
| I-D14 | LG7:10317470 | LG7:10318016 |
| I-D14 | LG7:730027 | LG7:735642 |
| I-D15 | LG1:12798788 | LG1:12800213 |
| I-D15 | LG10:11339577 | LG10:11399306 |
| I-D15 | LG10:11945378 | LG10:11946686 |
| I-D15 | LG10:6636919 | LG10:6637596 |
| I-D15 | LG11:12232894 | LG11:12233491 |
| I-D15 | LG11:3809129 | LG11:3810291 |
| I-D15 | LG12:2999876 | LG12:3000129 |
| I-D15 | LG12:6454355 | LG12:6455299 |
| I-D15 | LG14:4326036 | LG14:4328055 |
| I-D15 | LG15:6754650 | LG15:6756317 |
| I-D15 | LG17:1910386 | LG17:1910533 |
| I-D15 | LG18:880986 | LG18:881002 |
| I-D15 | LG3:6633229 | LG3:6685979 |
| I-D15 | LG4:10226043 | LG4:10226321 |
| I-D15 | LG5:3151198 | LG5:3151834 |
| I-D15 | LG5:8429621 | LG5:8431112 |
| I-D15 | LG6:9330232 | LG6:9332282 |
| I-D15 | LG7:11948989 | LG7:11950481 |
| I-D16 | LG1:1373467 | LG1:1610023 |
| I-D16 | LG1:4208636 | LG1:4208992 |
| I-D16 | LG10:11339577 | LG10:11399306 |
| I-D16 | LG10:13091621 | LG10:13093896 |
| I-D16 | LG10:5802642 | LG10:5803445 |
| I-D16 | LG11:12925515 | LG11:12927976 |
| I-D16 | LG12:6891314 | LG12:6891490 |
| I-D16 | LG14:5476032 | LG14:5476639 |
| I-D16 | LG16:1328390 | LG16:1329335 |
| I-D16 | LG17:1249394 | LG17:1250269 |
| I-D16 | LG2:10659371 | LG2:10661166 |
| I-D16 | LG2:5389498 | LG2:5440784 |
| I-D16 | LG2:912329 | LG2:914137 |
| I-D16 | LG3:7155425 | LG3:7859148 |
| I-D16 | LG3:9235725 | LG3:9236529 |
| I-D16 | LG4:11999231 | LG4:12000363 |
| I-D16 | LG5:8383192 | LG5:8383730 |
| I-D16 | LG6:226251 | LG6:226976 |
| I-D16 | LG7:914697 | LG7:916285 |
| I-D16 | LG9:7895489 | LG9:7896099 |
| I-D17 | LG1:1638050 | LG1:1650899 |
| I-D17 | LG1:8692213 | LG1:8692738 |
| I-D17 | LG10:8640649 | LG10:8641654 |
| I-D17 | LG11:7803251 | LG11:7807457 |
| I-D17 | LG12:7896962 | LG12:7898515 |
| I-D17 | LG16:936930 | LG16:937791 |
| I-D17 | LG17:1404045 | LG17:1404112 |
| I-D17 | LG2:2317071 | LG2:2447183 |
| I-D17 | LG3:5816803 | LG3:5817433 |
| I-D17 | LG3:7155425 | LG3:7859148 |
| I-D17 | LG4:3178502 | LG4:3242555 |
| I-D17 | LG6:320456 | LG6:322977 |
| I-D17 | LG7:6457254 | LG7:6460464 |
| I-D17 | LG9:14105254 | LG9:14114580 |
| I-D17 | LG9:5259878 | LG9:5260700 |
| I-D18 | LG10:11339577 | LG10:11399306 |
| I-D18 | LG10:2374074 | LG10:2376071 |
| I-D18 | LG11:17156341 | LG11:17176778 |
| I-D18 | LG11:9026696 | LG11:9027188 |
| I-D18 | LG14:2720383 | LG14:2780210 |
| I-D18 | LG14:3252249 | LG14:3254241 |
| I-D18 | LG14:4299897 | LG14:4312922 |
| I-D18 | LG15:7093324 | LG15:7094275 |
| I-D18 | LG16:2444409 | LG16:2495812 |
| I-D18 | LG17:1893744 | LG17:1894782 |
| I-D18 | LG18:857470 | LG18:858225 |
| I-D18 | LG2:7851999 | LG2:7853289 |
| I-D18 | LG2:912329 | LG2:914137 |
| I-D18 | LG3:4644794 | LG3:4645276 |
| I-D18 | LG3:6633229 | LG3:6685979 |
| I-D18 | LG3:9894868 | LG3:9895607 |
| I-D18 | LG4:3178502 | LG4:3242555 |
| I-D18 | LG5:5998516 | LG5:6001193 |
| I-D18 | LG6:8037625 | LG6:8038210 |
| I-D18 | LG9:13000771 | LG9:13001836 |
| I-D18 | LG9:7703540 | LG9:7705170 |
| I-D19 | LG1:2835895 | LG1:2837754 |
| I-D19 | LG1:8705333 | LG1:8706214 |
| I-D19 | LG10:4201693 | LG10:4202334 |
| I-D19 | LG11:1017266 | LG11:1017986 |
| I-D19 | LG11:14316439 | LG11:14320129 |
| I-D19 | LG12:3414334 | LG12:3416418 |
| I-D19 | LG14:2556077 | LG14:2558430 |
| I-D19 | LG16:2444409 | LG16:2495812 |
| I-D19 | LG3:11992213 | LG3:11992405 |
| I-D19 | LG3:7155425 | LG3:7859148 |
| I-D19 | LG4:12819195 | LG4:12821054 |
| I-D19 | LG4:813707 | LG4:869821 |
| I-D19 | LG6:11621430 | LG6:11623856 |
| I-D19 | LG7:2967082 | LG7:2968310 |
| I-D19 | LG9:4108477 | LG9:4108901 |
| I-D20 | LG1:9239774 | LG1:9240468 |
| I-D20 | LG10:11339577 | LG10:11399306 |
| I-D20 | LG11:2906876 | LG11:2909177 |
| I-D20 | LG12:4897107 | LG12:4897340 |
| I-D20 | LG14:2435337 | LG14:2436447 |
| I-D20 | LG16:2444409 | LG16:2495812 |
| I-D20 | LG17:3111959 | LG17:3114843 |
| I-D20 | LG2:4449767 | LG2:4455760 |
| I-D20 | LG3:7155425 | LG3:7859148 |
| I-D20 | LG3:8277312 | LG3:8277954 |
| I-D20 | LG4:5925431 | LG4:5925671 |
| I-D20 | LG4:813707 | LG4:869821 |
| I-D20 | LG5:3659086 | LG5:3660626 |
| I-D20 | LG7:12219877 | LG7:12220330 |
| I-D20 | LG7:9405774 | LG7:9408669 |
| I-D20 | LG8:4798066 | LG8:4798901 |
| I-D21 | LG1:5518422 | LG1:5569968 |
| I-D21 | LG1:5835355 | LG1:5837103 |
| I-D21 | LG1:7275048 | LG1:7332652 |
| I-D21 | LG10:6040353 | LG10:6040899 |
| I-D21 | LG11:17156341 | LG11:17176778 |
| I-D21 | LG14:2413473 | LG14:2414281 |
| I-D21 | LG15:3905143 | LG15:3906584 |
| I-D21 | LG17:2359180 | LG17:2494626 |
| I-D21 | LG17:2752915 | LG17:2754113 |
| I-D21 | LG18:795006 | LG18:795403 |
| I-D21 | LG2:5854784 | LG2:5855219 |
| I-D21 | LG3:6633229 | LG3:6685979 |
| I-D21 | LG4:1042706 | LG4:1044637 |
| I-D21 | LG4:2794502 | LG4:2860966 |
| I-D21 | LG4:3178502 | LG4:3242555 |
| I-D21 | LG6:2019190 | LG6:2019241 |
| I-D21 | LG6:9340868 | LG6:9342510 |
| I-D21 | LG7:7652087 | LG7:7654001 |
| I-D22 | LG1:1021647 | LG1:1140223 |
| I-D22 | LG1:1367334 | LG1:1368369 |
| I-D22 | LG1:1373467 | LG1:1610023 |
| I-D22 | LG1:5516835 | LG1:5518411 |
| I-D22 | LG1:5692482 | LG1:5696329 |
| I-D22 | LG1:7275048 | LG1:7332652 |
| I-D22 | LG10:11339577 | LG10:11399306 |
| I-D22 | LG11:11918318 | LG11:11918534 |
| I-D22 | LG14:2037910 | LG14:2038955 |
| I-D22 | LG15:7758407 | LG15:7770249 |
| I-D22 | LG16:2444409 | LG16:2495812 |
| I-D22 | LG17:345017 | LG17:346881 |
| I-D22 | LG2:8802633 | LG2:8803208 |
| I-D22 | LG3:12616617 | LG3:12617515 |
| I-D22 | LG3:6317081 | LG3:6318998 |
| I-D22 | LG3:6633229 | LG3:6685979 |
| I-D22 | LG4:7864504 | LG4:7864794 |
| I-D22 | LG4:813707 | LG4:869821 |
| I-D22 | LG5:1597889 | LG5:1598307 |
| I-D22 | LG6:2517552 | LG6:2517948 |
| I-D22 | LG7:1221441 | LG7:1222510 |
| I-D22 | LG7:5616172 | LG7:5617826 |
| I-D22 | LG8:5911906 | LG8:5913109 |
| I-D22 | LG9:7632557 | LG9:7634663 |
| II-D2 | LG1:10487323 | LG1:10487514 |
| II-D2 | LG11:13798696 | LG11:13799894 |
| II-D2 | LG12:11735019 | LG12:11735580 |
| II-D2 | LG12:5121550 | LG12:5122682 |
| II-D2 | LG15:6390772 | LG15:6391873 |
| II-D2 | LG18:264900 | LG18:267407 |
| II-D2 | LG2:12572030 | LG2:12573187 |
| II-D2 | LG2:496903 | LG2:497454 |
| II-D2 | LG3:6631412 | LG3:6632912 |
| II-D2 | LG4:1818112 | LG4:1818734 |
| II-D2 | LG4:2089772 | LG4:2862583 |
| II-D2 | LG4:3182518 | LG4:3242781 |
| II-D2 | LG5:465635 | LG5:466508 |
| II-D2 | LG7:13819757 | LG7:13820872 |
| II-D2 | LG8:8020877 | LG8:8021214 |
| II-D2 | LG9:9817976 | LG9:9819055 |
| II-D4 | LG1:15762193 | LG1:15763087 |
| II-D4 | LG1:5169878 | LG1:5172923 |
| II-D4 | LG10:11345659 | LG10:11398224 |
| II-D4 | LG10:12857429 | LG10:12857650 |
| II-D4 | LG10:2887258 | LG10:2888756 |
| II-D4 | LG10:5818275 | LG10:5820042 |
| II-D4 | LG11:13403128 | LG11:13404032 |
| II-D4 | LG11:8792869 | LG11:8794374 |
| II-D4 | LG12:1354890 | LG12:1356544 |
| II-D4 | LG13:6452209 | LG13:6454319 |
| II-D4 | LG14:5561379 | LG14:5561681 |
| II-D4 | LG14:6533128 | LG14:6584897 |
| II-D4 | LG14:6591426 | LG14:6643930 |
| II-D4 | LG15:6368006 | LG15:6369337 |
| II-D4 | LG16:1486974 | LG16:1487064 |
| II-D4 | LG2:1265778 | LG2:1266157 |
| II-D4 | LG2:9589109 | LG2:9590270 |
| II-D4 | LG3:166837 | LG3:167808 |
| II-D4 | LG3:6218670 | LG3:6220850 |
| II-D4 | LG4:3182518 | LG4:3242781 |
| II-D4 | LG4:814130 | LG4:870135 |
| II-D4 | LG5:2635097 | LG5:2636809 |
| II-D4 | LG5:9912544 | LG5:9912640 |
| II-D4 | LG6:11177111 | LG6:11179933 |
| II-D4 | LG6:1998010 | LG6:1999652 |
| II-D4 | LG6:5800538 | LG6:6528385 |
| II-D4 | LG7:528660 | LG7:530840 |
| II-D4 | LG9:14584475 | LG9:14584900 |
| II-D4 | LG9:1969447 | LG9:2393050 |
| II-D4 | LG9:4414810 | LG9:4416173 |
| II-D4 | LG9:534517 | LG9:536123 |
| II-D4 | LG9:7721801 | LG9:7722708 |
| II-D8 | LG10:10241191 | LG10:10242532 |
| II-D8 | LG11:14155552 | LG11:14732185 |
| II-D8 | LG11:15762899 | LG11:15764458 |
| II-D8 | LG12:11989006 | LG12:11989763 |
| II-D8 | LG13:1215543 | LG13:1275773 |
| II-D8 | LG13:8258961 | LG13:8259549 |
| II-D8 | LG14:6932427 | LG14:6933146 |
| II-D8 | LG15:5215167 | LG15:5215240 |
| II-D8 | LG18:679213 | LG18:680352 |
| II-D8 | LG2:9165511 | LG2:9167654 |
| II-D8 | LG3:13509879 | LG3:13513075 |
| II-D8 | LG3:1544240 | LG3:1545627 |
| II-D8 | LG3:6632962 | LG3:6684790 |
| II-D8 | LG3:7780425 | LG3:7851607 |
| II-D8 | LG4:8653535 | LG4:8654023 |
| II-D8 | LG5:2516074 | LG5:2517133 |
| II-D8 | LG5:5826696 | LG5:5827827 |
| II-D8 | LG6:3422714 | LG6:3422991 |
| II-D8 | LG7:3170352 | LG7:3171536 |
| II-D8 | LG8:875643 | LG8:876336 |
| II-D9 | LG1:16125243 | LG1:16127736 |
| II-D9 | LG11:1312191 | LG11:1313262 |
| II-D9 | LG11:7407163 | LG11:7407822 |
| II-D9 | LG12:11438061 | LG12:11439022 |
| II-D9 | LG12:4011030 | LG12:4013175 |
| II-D9 | LG15:1941064 | LG15:1941503 |
| II-D9 | LG2:4836360 | LG2:4838979 |
| II-D9 | LG3:10643990 | LG3:10644610 |
| II-D9 | LG3:6632962 | LG3:6684790 |
| II-D9 | LG3:7780425 | LG3:7851607 |
| II-D9 | LG4:7969407 | LG4:7971295 |
| II-D9 | LG5:2482546 | LG5:2483320 |
| II-D9 | LG5:7156070 | LG5:7157194 |
| II-D9 | LG7:1836535 | LG7:1836607 |
| II-D9 | LG8:3334920 | LG8:3335184 |
| II-D9 | LG8:4048843 | LG8:4818282 |
| II-D12 | LG1:10666322 | LG1:10666625 |
| II-D12 | LG1:4962110 | LG1:4964556 |
| II-D12 | LG10:1665855 | LG10:1667196 |
| II-D12 | LG10:6291747 | LG10:6294828 |
| II-D12 | LG11:4427355 | LG11:4428045 |
| II-D12 | LG13:1215543 | LG13:1275773 |
| II-D12 | LG13:2094672 | LG13:2096203 |
| II-D12 | LG13:4606975 | LG13:4665316 |
| II-D12 | LG13:7122890 | LG13:7123204 |
| II-D12 | LG16:2443198 | LG16:2495437 |
| II-D12 | LG18:866765 | LG18:867684 |
| II-D12 | LG2:7499381 | LG2:7502645 |
| II-D12 | LG3:14345412 | LG3:14346102 |
| II-D12 | LG3:3059489 | LG3:3060372 |
| II-D12 | LG3:6632962 | LG3:6684790 |
| II-D12 | LG3:7780425 | LG3:7851607 |
| II-D12 | LG4:11858789 | LG4:11859574 |
| II-D12 | LG4:814130 | LG4:870135 |
| II-D12 | LG5:4760317 | LG5:4761815 |
| II-D12 | LG7:14411724 | LG7:14412783 |
| II-D12 | LG8:6992579 | LG8:6994211 |
| II-D12 | LG9:6221305 | LG9:6222648 |
| II-D14 | LG1:14862084 | LG1:14863002 |
| II-D14 | LG10:8777555 | LG10:8780451 |
| II-D14 | LG11:525655 | LG11:528142 |
| II-D14 | LG11:8794402 | LG11:8794771 |
| II-D14 | LG12:7571754 | LG12:7572784 |
| II-D14 | LG15:3634103 | LG15:3634565 |
| II-D14 | LG16:2372482 | LG16:2372931 |
| II-D14 | LG18:1004585 | LG18:1005656 |
| II-D14 | LG2:12475854 | LG2:12478338 |
| II-D14 | LG2:2266518 | LG2:2267189 |
| II-D14 | LG4:13867574 | LG4:13868724 |
| II-D14 | LG4:3182518 | LG4:3242781 |
| II-D14 | LG4:814130 | LG4:870135 |
| II-D14 | LG4:9184506 | LG4:9184621 |
| II-D14 | LG5:3171703 | LG5:3172459 |
| II-D14 | LG6:6976765 | LG6:6977286 |
| II-D14 | LG7:1026687 | LG7:1029894 |
| II-D14 | LG8:1281101 | LG8:1281241 |
| II-D14 | LG8:7169061 | LG8:7171020 |
| II-D14 | LG9:12999247 | LG9:13000377 |
| II-D14 | LG9:1938185 | LG9:1940060 |
| II-D14 | LG9:1969447 | LG9:2393050 |
| II-D14 | LG9:6204208 | LG9:6206520 |
| II-D16 | LG10:802631 | LG10:1341133 |
| II-D16 | LG11:14155552 | LG11:14732185 |
| II-D16 | LG11:15020513 | LG11:15023549 |
| II-D16 | LG12:9084970 | LG12:9085776 |
| II-D16 | LG13:9469806 | LG13:9481427 |
| II-D16 | LG14:5394136 | LG14:5405319 |
| II-D16 | LG14:6533128 | LG14:6584897 |
| II-D16 | LG14:6589628 | LG14:6590470 |
| II-D16 | LG16:1424532 | LG16:1424838 |
| II-D16 | LG2:374681 | LG2:374887 |
| II-D16 | LG2:6682080 | LG2:7357392 |
| II-D16 | LG3:6632962 | LG3:6684790 |
| II-D16 | LG3:7780425 | LG3:7851607 |
| II-D16 | LG4:13230541 | LG4:13230998 |
| II-D16 | LG5:10846317 | LG5:10846508 |
| II-D16 | LG5:2613979 | LG5:2615260 |
| II-D16 | LG6:11725116 | LG6:11726489 |
| II-D16 | LG9:10636059 | LG9:10636305 |
| II-D17 | LG1:5518755 | LG1:5697008 |
| II-D17 | LG1:6830784 | LG1:6830956 |
| II-D17 | LG1:7274995 | LG1:8183476 |
| II-D17 | LG11:3003562 | LG11:3003920 |
| II-D17 | LG12:12235684 | LG12:12236366 |
| II-D17 | LG12:4395908 | LG12:4396615 |
| II-D17 | LG13:5669156 | LG13:5669454 |
| II-D17 | LG15:2380260 | LG15:2380468 |
| II-D17 | LG15:7447359 | LG15:7448295 |
| II-D17 | LG16:2443198 | LG16:2495437 |
| II-D17 | LG18:525524 | LG18:528290 |
| II-D17 | LG2:11299765 | LG2:11299857 |
| II-D17 | LG2:8426773 | LG2:8426840 |
| II-D17 | LG3:4032278 | LG3:4032942 |
| II-D17 | LG3:6632962 | LG3:6684790 |
| II-D17 | LG3:7780425 | LG3:7851607 |
| II-D17 | LG4:10788000 | LG4:10788073 |
| II-D17 | LG5:6344771 | LG5:6346289 |
| II-D17 | LG6:2877284 | LG6:2878254 |
| II-D17 | LG7:11351736 | LG7:11354709 |
| II-D17 | LG7:213199 | LG7:214459 |
| II-D17 | LG9:1969447 | LG9:2393050 |
| II-D17 | LG9:2400904 | LG9:2455337 |
| II-D17 | LG9:3548057 | LG9:3549322 |
| II-D21 | LG11:901747 | LG11:902292 |
| II-D21 | LG12:10437007 | LG12:10828348 |
| II-D21 | LG13:4543792 | LG13:4547503 |
| II-D21 | LG13:4552323 | LG13:4603338 |
| II-D21 | LG14:10276475 | LG14:10278140 |
| II-D21 | LG14:9393715 | LG14:10209912 |
| II-D21 | LG15:4629704 | LG15:4630494 |
| II-D21 | LG17:844542 | LG17:846598 |
| II-D21 | LG2:3994471 | LG2:3997934 |
| II-D21 | LG2:8554853 | LG2:8554987 |
| II-D21 | LG2:975672 | LG2:979619 |
| II-D21 | LG3:6143240 | LG3:6144414 |
| II-D21 | LG4:3182518 | LG4:3242781 |
| II-D21 | LG5:11232151 | LG5:11233167 |
| II-D21 | LG5:2765505 | LG5:2766827 |
| II-D21 | LG6:4310499 | LG6:4310836 |
| II-D21 | LG7:1100742 | LG7:1100891 |
| II-D21 | LG7:15575698 | LG7:15576619 |
| II-D21 | LG9:4072696 | LG9:4073647 |
| II-D27 | LG1:5169229 | LG1:5169858 |
| II-D27 | LG1:9412970 | LG1:9413013 |
| II-D27 | LG10:8987667 | LG10:8989062 |
| II-D27 | LG12:10317335 | LG12:10318671 |
| II-D27 | LG12:4658897 | LG12:4659095 |
| II-D27 | LG14:7529121 | LG14:7529295 |
| II-D27 | LG15:2176790 | LG15:2177217 |
| II-D27 | LG17:2404388 | LG17:2406038 |
| II-D27 | LG18:295297 | LG18:296825 |
| II-D27 | LG4:9049086 | LG4:9049865 |
| II-D27 | LG6:6637917 | LG6:6638380 |
| II-D27 | LG7:16574230 | LG7:16574667 |

**Table S3. Number of crossover events defined by different tract length.**

|  | Tract length | | |
| --- | --- | --- | --- |
|  | ≥500 kb | ≥100 kb | ≥10 kb |
| No. of COs detected | 531 | 554 | 606 |
| No. of COs per drone | 17 | 17 | 19 |
| cM/Mb | 7.7 | 8.0 | 8.7 |

**Table S4. Raw correlations between recombination rate and GC-content, heterozygosity, gene density and exon percentage at different scale.** The genome is divided into 10 kb (100 kb, 200 kb and 500kb) non-overlapping windows. The GC-content (percentage of identifiable bases that are G or C), heterozygosity (heterozygosity between the two haplotypes from the same colony, averaged for two colonies in this study), gene density (number of genes per bp of sequence) and exon percentage (span of exonic coding sequence per base) are calculated for each window. The raw correlations between these variables at each window size are shown in table below.

| Variables | Block size | Spearman’s rho | *p*-value |
| --- | --- | --- | --- |
| GC- content  and  Recombination rate | 10 kb | 0.035 | 3.0e-07 |
|  | 100 kb | 0.082 | 1.4e-04 |
|  | 200 kb | 0.101 | 8.0e-04 |
|  | 500 kb | 0.125 | 0.0087 |
| Recombination rate  and  Heterozygosity | 10 kb | 0.067 | <2.2e-16 |
|  | 100 kb | 0.161 | 3.6e-14 |
|  | 200 kb | 0.241 | 6.4e-16 |
|  | 500 kb | 0.350 | 3.3e-14 |
| Gene density  and  Recombination rate | 10 kb | -0.033 | 1.5e-06 |
|  | 100 kb | -0.066 | 0.0022 |
|  | 200 kb | -0.088 | 0.0037 |
|  | 500 kb | -0.088 | 0.066 |
| Exon percentage  and  Recombination rate | 10 kb | -0.031 | 5.3e-06 |
|  | 100 kb | -0.066 | 0.0019 |
|  | 200 kb | -0.075 | 0.013 |
|  | 500 kb | -0.076 | 0.109 |
| Gene density  and  Heterozygosity | 10 kb | -0.304 | <2.2e-16 |
|  | 100 kb | -0.401 | <2.2e-16 |
|  | 200 kb | -0.380 | <2.2e-16 |
|  | 500 kb | -0.281 | 1.7e-09 |
| Exon percentage  and  Heterozygosity | 10 kb | -0.334 | <2.2e-16 |
|  | 100 kb | -0.409 | <2.2e-16 |
|  | 200 kb | -0.376 | <2.2e-16 |
|  | 500 kb | -0.265 | 1.5e-08 |

**Table S5. Partial correlations between recombination rate and heterozygosity, between gene density and heterozygosity and between exon percentage and heterozygosity.** Partial correlations are calculated by ‘ppcor’ package in R, using Spearman’s method.

| Variables | Control | Block size | Partial correlation | *p*-value |
| --- | --- | --- | --- | --- |
| Recombination rate  and  Heterozygosity | Gene density | 10 kb | 0.060 | 9.6e-19 |
|  |  | 100 kb | 0.148 | 4.3e-12 |
|  |  | 200 kb | 0.225 | 4.7e-14 |
|  |  | 500 kb | 0.340 | 1.9e-13 |
| Recombination rate  and  Heterozygosity | Exon percentage | 10 kb | 0.060 | 7.1e-19 |
|  |  | 100 kb | 0.147 | 4.9e-12 |
|  |  | 200 kb | 0.230 | 1.3e-14 |
|  |  | 500 kb | 0.343 | 1.2e-13 |
| Gene density  and  Heterozygosity | Recombination rate | 10 kb | -0.303 | 0 |
|  |  | 100 kb | -0.397 | 5.1e-83 |
|  |  | 200 kb | -0.371 | 4.4e-37 |
|  |  | 500 kb | -0.269 | 9.7e-09 |
| Exon percentage  and  Heterozygosity | Recombination rate | 10 kb | -0.332 | 0 |
|  |  | 100 kb | -0.404 | 2.2e-86 |
|  |  | 200 kb | -0.370 | 9.6e-37 |
|  |  | 500 kb | -0.255 | 5.5e-08 |
